# Weight Loss Barriers and Dietary Quality of Intermittent and Continuous Dieters in Women with a History of Gestational Diabetes

**DOI:** 10.3390/ijerph181910243

**Published:** 2021-09-29

**Authors:** Kristy L. Gray, Peter M. Clifton, Jennifer B. Keogh

**Affiliations:** 1 UniSA, Clinical and Health Sciences, University of South Australia, Adelaide, SA 5001, Australia; Peter.Clifton@unisa.edu.au (P.M.C.); jennifer.keogh@unisa.edu.au (J.B.K.); 2 Alliance for Research in Exercise, Nutrition and Activity (ARENA), University of South Australia, Adelaide, SA 5001, Australia

**Keywords:** diabetes prevention, gestational diabetes, weight loss, women, intermittent energy restriction, intermittent fasting, theoretical domains framework, weight-loss barriers

## Abstract

Weight-loss after gestational diabetes (GDM) lowers the risk of type-2 diabetes (T2DM). Intermittent energy restriction (IER) produces comparable weight-loss to continuous energy restriction (CER), but long-term adherence remains difficult in this population. This exploratory secondary analysis of a 12-month trial comparing IER to CER following GDM examined weight-loss and dietary quality associated with barriers to weight-loss or T2DM risk perception as assessed in a Likert scale questionnaire at baseline. The participants had a median (IQR) BMI of 32.6 (9.4) kg/m^2^ and 3 (4) years postpartum (*n* = 121). Forty-five percent (*n* = 54) of the participants thought they were at a high risk of developing T2DM. Greater affordability of healthy food was related with greater weight-loss at 3 months (*p* = 0.044, *n* = 85). At 12 months, there was no significant relationship between weight-loss and the barriers to weight-loss (*p* > 0.05). CER had superior improvement in dietary quality at 12 months (CER 11 ± 10, IER 6 ± 5.6, *n* = 42, *p* = 0.05). Under the Theoretical Domains Framework, the barriers were predominantly related to behavioral regulation (*n* = 83, 69%; *n* = 76, 63%) and environmental context and resources (*n* = 67, 56%). Interventions for diabetes prevention in this population should include behavioral regulation strategies, consider the family home environment, and ensure that the risk of T2DM is conveyed. Women choosing IER may benefit from education to improve their dietary quality.

## 1. Introduction

Women with a history of gestational diabetes (GDM) have a nearly 10-fold risk of developing type-2 diabetes (T2DM) compared with women with no GDM in pregnancy, making GDM one of the highest single risk factors for T2DM development [1]. Despite this, research shows that women with previous GDM typically do not perceive themselves to be at a high risk for T2DM development [2,3,4,5]. Women with a history of GDM who are overweight can significantly reduce their risk of developing T2DM through lifestyle changes resulting in weight-loss [6,7]. However, they face multifaceted barriers to achieving weight-loss, and family responsibilities interfering with weight-loss attempts and motivation are often at the forefront of this [2,8,9].

Intermittent energy restriction (IER) has become a popular weight-loss strategy in recent years and may offer more flexibility in eating over the week compared with a continuous diet [10,11]. Research to date suggests that IER can achieve comparable but not superior weight loss and metabolic improvements to continuous energy restriction (CER), but long-term adherence does not appear to be improved in intermittent dieters compared with continuous dieters [10,12,13]. The results from our 12-month randomized control trial (RCT) investigating the effect of IER on weight-loss and diabetes risk markers in women with previous GDM showed that IER can produce comparable weight-loss and metabolic improvements. However, there was close to 50% attrition, which limited the conclusions that could be drawn from the study [14]. Low participation and high dropout in this population have been previously reported, highlighting poor long-term adherence to lifestyle changes in women with previous GDM [15,16,17,18].

Understanding the barriers to weight-loss assists with successful intervention development by identifying individual characteristics that may help or hinder weight-loss efforts and adherence to a diabetes prevention program [19]. Interventions aiming to improve health outcomes in women with a history of GDM need to consider these individual characteristics that may predict health-related behaviors and encompass individual needs into such interventions [17,20]. Additionally, the development of interventions that require behavior changes should be underpinned by an appropriate behavior change theory or framework [21]. The Theoretical Domains Framework (TDF) incorporates theories of behavior change into one integrative framework and allows researchers to classify the determinants of a behavior to inform implementation [22,23]. This paper reports the results from an exploratory secondary analysis of our 12-month clinical trial. The results from the primary outcomes of the clinical trial have been published elsewhere [14]. Here, we use the TDF integrated into the Capability, Opportunity, Motivation-Behavior model (COM-B model) [23] to examine whether the weight-loss of the participants in a 12-month clinical trial investigating IER compared to CER in women with previous GDM was associated with barriers to weight-loss and perception of the risk of T2DM at baseline. Furthermore, we present dietary quality data from the clinical trial and investigate how barriers to weight-loss and the perception of diet risk may have influenced the overall diet quality in 12-month IER and CER weight-loss intervention in women with a history of GDM. 

## 2. Materials and Methods

### 2.1. Participants and Study Design

The participants were enrolled in a 12-month RCT between March 2018 and March 2019 which investigated IER as an alternative diet strategy to CER. The study design and recruitment processes have been previously reported [14]. Briefly, the participants were females aged ≥18 years with previous GDM, no diagnosis of any other type of diabetes and a BMI ≥25 kg/m^2^. The participants were randomized 1:1 to either an IER (500 kcal (2092 kJ) per day; 40% protein, 35% carbohydrate and 25% fat with 7.5% saturated fat for 2 non-consecutive days each week) or CER diet (1500 kcal (6276 kJ) per day; 30% protein, 45% carbohydrate and 25% fat with 7.5% saturated fat for 7 days a week). Both diets provided approximately 25% energy restriction per week. The participants were asked to complete diet checklists for 2 days a week in the month leading up to their clinic appointments, and the results of their dietary intake were published [14]. Randomization was achieved using an online random number generator (www.randomization.com, accessed on 1 August 2021) and was stratified by the number of years since GDM (≤5 years and >5 years) and BMI (<30 kg/m^2^ and ≥30 kg/m^2^).

### 2.2. Measurements

Height was measured at the baseline visit without shoes on using a wall-mounted stadiometer (SECA, Hamburg, Germany) and recorded to the nearest 0.1 cm. Weight was measured at baseline, 3 and 12 months after an overnight fast and in light clothing without shoes using calibrated electronic digital scales (SECA, Hamburg, Germany) to the nearest 0.1 kg. The BMI was calculated from the height and weight using the equation BMI = [weight(kg)/height(m)^2^].

Participants were asked to complete a questionnaire at their baseline visit which included questions about barriers to weight-loss and the perception of risk of developing T2DM. The barriers to weight-loss questionnaire listed 10 items and asked participants to respond on a 5-point Likert scale (“Strongly agree”, “Agree”, “Not sure”, “Disagree” or “Strongly disagree”). Space for open text comments was provided for participants to list any other barriers to weight-loss. The participants were also asked if they thought they were at risk of developing T2DM and responded on a 4-point scale (“Yes, high risk”, “Yes, moderate risk”, “Yes, low risk” or “No, I am not at risk”). The questions were linked to the Theoretical Domains Framework (TDF). All the items in the questionnaire underwent expert review for content validation and were used in our previous survey of women in Australia with a history of gestational diabetes [2,24]. The questionnaire items are listed in Table 1. Dietary quality was assessed at baseline and after 12 months using the Commonwealth Scientific and Industrial Research Organisation (CSIRO) Healthy Diet Score (HDS). This is a validated online dietary quality assessment tool that includes questions regarding the quantity, quality and variety of an individual’s diet. The scoring algorithm compares the intake across the different food groups as well as discretionary intake against the Australian Dietary Guidelines (ADGs), with each food group being assigned a maximum of 10 points and discretionary intake being assigned a maximum of 20 points [25]. Upon completion, the results are immediately displayed as a score, with a maximum score of 100. A higher score represents greater compliance with the ADGs, and a lower score indicates a lower quality diet. The users do not receive information regarding their score from each of the food groups or discretionary categories, but three personalized suggestions on how they could improve their diet, such as lowering discretionary intake, including more wholegrains or consuming more vegetables, are provided with the score.

### 2.3. Statistical Analysis

The data were analyzed using IBM SPSS Statistical Software version 26 for Windows (IBM, Chicago IL, USA). Significance was set at *p* < 0.05. The data were tested for normality using Q-Q plots, histograms and Shapiro–Wilk tests. The results for categorical variables were presented as numbers (%). Results for the continuous variables were presented as the mean ± SD for normally distributed data and the median (IQR) for non-normal data. The results from the 5-point Likert scales were collated to a 3-point scale by grouping “Agree” and “Strongly agree” responses and “Disagree” and “Strongly disagree” responses together for the descriptive analysis. Independent t-tests were used to determine the differences between the groups. For the association analysis, the results from the barriers to weight-loss were collated to a 2-point scale (“Agree” and “Disagree”), omitting the “Not sure” responses. Spearman’s correlation was used to determine the associations with the ordinal variables and the dependent variables. Variables found to be correlated to the dependent variable were entered into Kruskal–Wallis non-parametric tests as the data were not normally distributed. Intention-to-treat analyses were run for the weight loss associations that were significant in the completers’ analysis using the last weight carried forward.

Each barrier was linked to a previously determined TDF domain. Open text comments (*n* = 32) for the barriers to weight-loss were categorized into the domains of the TDF (*n* = 37) by the primary researcher (K.L.G.). Supplement S1 shows the TDF and COM-B model. 

This was an exploratory secondary analysis of a 12-month clinical trial; therefore, no power calculation was undertaken to determine a required sample size for the variables reported here, and no correction for multiple testing was performed.

## 3. Results

One hundred twenty-one participants were randomized to the clinical trial, and 62 participants completed the trial to 12 months (49% attrition). Withdrawal was similar between both diet groups (IER 48% (*n* = 29), CER 50% (*n* = 30), *p* = 0.8). One participant withdrew at the baseline appointment after randomization and did not complete the questionnaire. There were 120 responses for the perception of diabetes risk question and 9 of the 10 barriers to weight-loss items measured at baseline. Three participants did not provide an answer to the barrier “It is easy to lose weight” (*n* = 117). The demographics, weight loss and diabetes marker outcome results were previously reported [14]. Briefly, the participants were predominantly in the obese weight category with a median (IQR) BMI of 32.6 (9.4) kg/m^2^, had a median (IQR) age of 40 (9) years and were 3 (4) years postpartum at baseline (Table 2). Weight-loss was statistically significant over time (*p* < 0.001) but not over time by diet group at 12 months (IER −4.8 ± 5.0 kg, CER −3.2 ± 5.0 kg, *p* = 0.17, *n* = 62).

### 3.1. Barriers to Weight-Loss and the Perception of Diabetes Risk

The most common barriers to weight-loss from the list in the questionnaire were “finding it hard to stay on a diet” (*n* = 83, 69%) (TDF domain: behavioral regulation), “finding it hard to deal with hunger while on a diet” (*n* = 76, 63%) (TDF domain: behavioral regulation) and “family responsibilities taking priority over weight-loss“ (*n* = 67, 56%) (TDF domain: environmental context and resources). Ninety-three percent (*n* = 112) of participants agreed that their family would support them to lose weight (TDF domain: social influences) and that they were motivated to lose weight (TDF domain: beliefs about capabilities). There were no significant differences between the diet groups and barriers to weight loss (*p* > 0.05). Table 1 shows the number and percentage of participants who responded with “Agree”, “Not sure” or “Disagree” to each item. Close to half of the participants reported that they thought they were at high risk for developing T2DM (TDF domain: beliefs about consequences) (*n* = 54, 45%), 39% (*n* = 47) answered moderate risk, 14% (*n* = 17) answered low risk, and 2% (*n* = 2) answered that they did not think they were at risk for developing T2DM (Table 1). There were no significant differences between IER and CER and the perceived risk of developing diabetes (*p* = 0.96). There were no significant correlations with the perception of diabetes risk or barriers to weight-loss between completers and non-completers (*p* > 0.05, *n* = 120).

Thirty-two comments were received regarding other barriers to weight loss in the baseline questionnaire, which were coded into 37 domains within the TDF. “Environmental context and resources” (*n* = 11), “beliefs about capabilities” (*n* = 8) and “social influences” (*n* = 8) were the most commonly allocated domains. Four comments were allocated into the domain “emotion”, while “memory, attention and decision processes” received three comments, “skills” received two comments, and “reinforcement” received one comment. Supplement S2 shows the comments received regarding barriers to weight-loss.

### 3.2. Barriers to Weight-Loss: Perception of Diabetes Risk and Weight-Loss

In a Spearman’s correlation matrix, only 1 of the 10 barriers was correlated with weight loss at 3 months (*n* = 85) (“I can’t afford to buy healthy foods”, *p* = 0.043, TDF domain: environmental context and resources). When entered into a Kruskal–Wallis test, being able to afford to buy healthy foods was related to more weight loss at 3 months in the completers’ analysis (*p* = 0.044, *n* = 85), and this remained significant in an intention-to-treat model (*p* = 0.009, *n* = 111). There were no significant correlations to weight-loss at 12 months with barriers to weight-loss (*p* > 0.05). The perception of diabetes risk was not correlated with weight loss at 3 months (*p* = 0.835, *n* = 89) or 12 months (*p* = 0.369, *n* = 62).

### 3.3. CSIRO Healthy Diet Score and Weight-Loss Outcomes

Ninety-eight CSIRO Healthy Diet Scores (HDS) were completed at baseline. The results were missing from *n* = 23 participants due to difficulties using the website, forgetting to complete it or withdrawing from the study after the first visit and not returning a completed CSIRO HDS. The mean CSIRO HDS at baseline was 56 ± 10. There was no significant difference in the CSIRO HDS between IER (mean 56 ± 9, *n* = 50) and CER (55 ± 11, *n* = 48) at baseline (*p* = 0.47). The baseline CSIRO score was not correlated with weight loss at three (*p* = 0.18, *n* = 89) or 12 months (*p* = 0.24, *n* = 62).

Forty-four of the 62 completers returned the CSIRO HDS at 12 months. The results were missing from *n* = 18 participants due to difficulties using the tool, not being able to complete the tool at baseline or forgetting to complete it. The mean CSIRO HDS at 12 months was 64 ± 10 (IER 62 ± 9, *n* = 25; CER 65 ± 12, *n* = 19, *p* = 0.34). The CSIRO scores improved between 0 and 12 months by 8 ± 6 points. The CER group showed a weak statistically significant improvement compared with the IER group in the CSIRO HDS between 0 and 12 months (CER 11 ± 10, IER 6 ± 5.6, *n* = 42, *p* = 0.05). 

### 3.4. Barriers to Weight-Loss: Perception of Diabetes Risk and CSIRO Healthy Diet Score

There were no significant correlations between the baseline HDS and barriers to weight-loss (*p* > 0.05). There were no responders who completed the HDS at 12 months who disagreed to the statement “I am motivated to lose weight” (*n* = 41 agreed). The statement “Dieting doesn’t work with my family meal schedule” was correlated with change in the HDS between the baseline and 12 months (*p* = 0.035, *n* = 31). When entered into a Kruskal–Wallis test, agreement to this statement was associated with a larger improvement in HDS scores between 0 and 12 months (*p* = 0.038, *n* = 31). No other barriers to weight-loss, perception of T2DM risk or diet group were significantly correlated with the HDS at baseline or at 12 months. There were no correlations between the barriers to weight-loss, perception of diabetes risk, diet group or change in the HDS over 12 months.

## 4. Discussion

This exploratory secondary analysis of a 12-month RCT investigating IER compared to CER in women with previous GDM found several relationships between the barriers to weight-loss at baseline, weight-loss and changes in dietary quality. We found no differences in the barriers to weight-loss and weight-loss success between the intermittent and continuous dieters. However, we did find that the CER group had better improvement in their dietary quality between the baseline and 12 months than the IER group. The barriers to weight-loss were centered around behavioral regulation and environmental context and resources, which was apparent despite high levels of self-reported motivation and family support.

Not being able to afford to buy healthy foods was associated with weight-loss at 3 months in this study. However, this association was lost at 12 months. Due to the small sample size, we were unable to draw any firm conclusions from this finding. Furthermore, only six completers in the study agreed they could not afford to buy healthy foods. The affordability of food varies greatly in different areas [26], and the population in the current study was largely living in a metropolitan location in Adelaide, South Australia, where food is accessible and more affordable than in rural locations. Other studies have shown that women with previous GDM report affordability of healthy foods as a barrier to weight loss [27]. In our study there were no correlations with the IER and CER diet groups in any of the models with barriers to weight-loss. However, the high attrition rate in our study is a limitation in our results, and given that affordability of healthy food is a well-known social determinant of health [28], further research investigating the affordability of an intermittent diet compared with a continuous diet would be valuable. Our analysis also showed that the participants who reported that dieting did not work with their family mealtimes at baseline had greater improvement in their diet quality scores at 12 months. This question only related to mealtimes and did not assess how participants felt about limiting discretionary snacks or changing meals they ate outside of the family environment. The HDS scoring algorithm has higher weighting for discretionary intake; each food group is assigned a maximum of 10 points, and discretionary intake is assigned a maximum of 20 points, with better adherence to the Australian Dietary Guidelines receiving a higher score in each group [25]. Therefore, removing indulgent foods independent of family meals will result in HDS improvement.

From the participants’ comments, the most common barriers to weight-loss at baseline in this cohort were related to behavioral regulation within the TDF. Environmental context and resources were also evident as a weight-loss barrier, with over half of the participants agreeing that their family responsibilities took priority over their weight-loss. Together, these findings outline a perceived lack of opportunity and capability to make the behavioral changes required for weight-loss. These findings are similar to our large observational survey of barriers to weight-loss in women with previous GDM, which found that almost two-thirds of the participants gave priority to their family responsibilities over weight-loss and over half reporting behavioral regulation as a key barrier [2].

The high withdrawal rate in this clinical trial limits the conclusions that can be drawn from our results. However, low levels of engagement and high attrition rates are common in this population [18], and we believe our study provides valuable results in the field which would benefit from further investigation with larger numbers. Additionally, our study design utilized a validated framework (TDF and COM-B model) to classify the barriers to weight-loss into determinants of behavior, which will help to inform the implementation of future interventions.

Another limitation to consider is that the questionnaire only listed 10 statements regarding barriers to weight-loss, and of the 120 women who completed the questionnaire, only 32 added comments regarding their own barriers to weight-loss. There may be other barriers to weight-loss that were not presented in the questionnaire list or comments. A more extensive questionnaire or focus groups could help to identify other weight-loss barriers in future studies. However, our findings suggest that behavioral regulation strategies and working around the limitations of the family environment setting are key to weight-loss success in this population. These findings are consistent with our previous research, which surveyed 429 women with previous GDM using the same questions [2], as well as a recent meta-analysis of strategies to improve healthy eating behaviors after pregnancy, which showed that interventions encompassing strategies to improve behavioral regulation and goal setting are more effective in this population than information provision aimed at increasing nutritional knowledge [17]. Together, these results reinforce evidence highlighting the need for interventions to be structured around the family when implementing health-related behavior change strategies in a family home setting with children [29].

The CER group in this study had a significantly improved CSIRO HDS score at 12 months compared with the IER group. This is consistent with the findings from an earlier study in which the Healthy Eating Index improved in the CER group compared with the IER group [30]. Weight-loss for IER in the primary results of this clinical trial was comparable to CER at 12 months (IER −4.8 ± 5.0 kg, CER −3.2 ± 5.0 kg, *p* = 0.2, *n* = 62) [14]. However, the CSIRO HDS scores suggest that despite comparable weight-loss, CER may result in better dietary improvements in the long term. This may be because dietary changes are required 7 days a week in a CER diet compared with only 2 days in an IER diet and help healthy eating habits to be developed. Our results suggest that for women with previous GDM choosing an IER diet for weight-loss and diabetes prevention in the long term, it may be beneficial to include education on healthy, unrestricted eating for non-fasting days to improve diet quality once a stable IER diet has been established. However, given the high attrition and small numbers in the study, our results do need to be interpreted with caution, and larger long-term studies which include a more comprehensive dietary quality assessment would be beneficial. The CSIRO HDS was chosen as it is a relatively quick and easy form of assessment, creating less burden on the participant than a food frequency questionnaire. However, it does not provide specific information on the intake of food groups or nutrients [25].

## 5. Conclusions

The findings from this study add insight to the results of our clinical trial, suggesting that despite self-reported high motivation, long-term weight-loss is difficult, and women with previous GDM face multiple barriers to weight-loss which are centered around the family environment and behavioral regulation. Furthermore, in the long term, women choosing an intermittent diet for weight-loss may benefit from an intervention that has a focus on improving dietary quality on non-fasting days. The high dropout rate in this study presents a serious limitation for interpreting the results, and larger studies are needed to confirm our findings. However, our findings are consistent with other research and suggest that interventions for diabetes prevention in women with previous GDM need to ensure that the risk of future T2DM is conveyed, that strategies to improve behavioral regulation are included and that the difficulties encompassing dietary change in the family home setting are considered. Qualitative research investigating the barriers and motivators for weight-loss in women with previous GDM following an IER or CER diet would provide further insight to assist with behavior change interventions.

## Figures and Tables

**Table 1:** Barriers to weight-loss and the perception of future diabetes risk at baseline in a randomized control trial investigating IER in women with previous GDM ^1^.

	All (*n* = 120)	IER (*n* = 61)	CER (*n* = 59)	*p* Value
My family will support me to lose weight (*n*) (%)				0.89
- Agree or strongly agree	112 (93)	58 (95)	54 (92)	
- Not sure	7 (6)	3 (5)	4 (7)	
- Disagree or strongly disagree	1 (0)	0 (0)	1 (2)	
It is easy to lose weight * (*n*) (%)				0.81
- Agree or strongly agree	9 (8)	6 (10)	3 (5)	
- Not sure	11 (9)	4 (7)	7 (12)	
- Disagree or strongly disagree	97 (83)	48 (83)	49 (83)	
My family responsibilities take priority over my weight (*n*) (%)				0.84
- Agree or strongly agree	67 (56)	34 (56)	33 (56)	
- Not sure	24 (20)	13 (21)	11 (19)	
- Disagree or strongly disagree	29 (24)	14 (23)	15 (25)	
I am motivated to lose weight (*n*) (%)				0.67
- Agree or strongly agree	112 (93)	58 (95)	54 (92)	
- Not sure	4 (3)	1 (2)	3 (5)	
- Disagree or strongly disagree	4 (3)	2 (3)	2 (3)	
Dieting doesn’t work with my family meal schedule (*n*) (%)				0.93
- Agree or strongly agree	16 (13)	8 (13)	8 (14)	
- Not sure	29 (24)	15 (25)	14 (24)	
- Disagree or strongly disagree	75 (63)	38 (63)	37 (63)	
I can’t afford to buy healthy foods (*n*) (%)				0.16
- Agree or strongly agree	6 (5)	2 (3)	4 (7)	
- Not sure	9 (8)	3 (5)	6 (10)	
- Disagree or strongly disagree	105 (88)	56 (92)	49 (83)	
I don’t have time to prepare healthy meals (*n*) (%)				0.10
- Agree or strongly agree	18 (15)	8 (13)	10 (17)	
- Not sure	17 (14)	7 (11)	10 (17)	
- Disagree or strongly disagree	85 (71)	46 (75)	39 (66)	
I am too tired to try and lose weight right now (*n*) (%)				0.17
- Agree or strongly agree	14 (12)	4 (66)	10 (17)	
- Not sure	12 (10)	9 (15)	3 (5)	
- Disagree or strongly disagree	94 (78)	48 (79)	46 (78)	
I find it hard to stay on a diet (*n*) (%)				0.44
- Agree or strongly agree	83 (69)	43 (70)	40 (68)	
- Not sure	19 (16)	10 (16)	9 (15)	
- Disagree or strongly disagree	18 (15)	8 (13)	10 (17)	
It is hard to deal with hunger while on a diet (*n*) (%)				0.73
- Agree or strongly agree	76 (63)	41 (67)	35 (59)	
- Not sure	14 (12)	6 (10)	8 (14)	
- Disagree or strongly disagree	30 (25)	14 (23)	16 (27)	
Do you think you are at risk of developing T2DM? (*n*) (%)				0.96
- Yes, high risk	54 (45)	29 (48)	25 (42)	
- Yes, moderate risk	47 (39)	21 (34)	26 (44)	
- Yes, low risk	17 (14)	10 (16)	7 (12)	
- No, I am not at risk	2 (2)	1 (2)	1 (2)	

* *n* = 117 for all responders, *n* = 58 for IER responders (*n* = 3 did not complete this question). CER: continuous energy restriction; ^1^ GDM: gestational diabetes; IER: intermittent energy restriction; T2DM: type-2 diabetes mellitus.

**Table 2:** Baseline characteristics of participants ^1^.

Characteristic	All Participants (*n* = 121)	IER (*n* = 61)	CER (*n* = 60)	*p* Value
Age (y)	39.6 (9.0)	39.3 (8.9)	40.2 (9.2)	0.75
Years postpartum (y)	2.9 (4.2)	2.4 (5.4)	3.1 (4.0)	0.81
Times had GDM (*n*)	1.0 (1)	1.0 (1)	1.0 (0)	0.77
GDM managed (*n*) (%)				0.96
- Diet	65 (54.2)	33 (53.2)	32 (55.2)	
- Metformin	12 (10)	6 (9.7)	6 (10.3)	
- Insulin	43 (35.8)	23 (37.1)	20 (34.5)	
Children (*n*)	2.0 (0)	2 (0)	2.0 (1.0)	0.43
Weight (kg)	89.9 (27.1)	90.3 (26.7)	87.0 (21.9)	0.12
BMI (kg/m^2^)	32.6 (9.4)	34.8 (9.6)	32.6 (8.4)	0.19
HbA1c (%)	5.4 (0.6)	5.4 (0.4)	5.3 (0.4)	0.24
Fasting glucose, plasma (mmol/L)	5.5 (0.5)	5.5 (0.6)	5.5 (0.5)	0.74

^1^ Data are available for IER *n* = 61, CER = 60 for all variables except HbA1c (IER *n* = 61, CER *n* = 59) and fasting plasma glucose (IER *n* = 51, CER *n* = 54). HbA1c and fasting finger-prick glucose were normally distributed and are displayed as means (SD). All other variables were not normally distributed and are shown as medians (IQR). CER: continuous energy restriction; GDM: gestational diabetes; IER: intermittent energy restriction.

## Data Availability

The data presented in this study are available on request from the corresponding author, subject to approval from the University of South Australia’s Human Research Ethics Committee.

## Supplementary Materials

The following are available online at https://www.mdpi.com/article/10.3390/ijerph181910243/s1. Supplement S1: The Theoretical Domains Framework integrated into the COM-B model (adapted from Cane et al. 2012, pg. 15 [22]); Supplement S2: Participant comments on barriers to weight loss at baseline visit commencing 12-month RCT, linked to the TDF and COM-B model.
