# Supplementary material for: Weight Loss Barriers and Dietary Quality of Intermittent and Continuous Dieters in Women with a History of Gestational Diabetes
Source: Int J Environ Res Public Health. 2021 Sep 29;18(19):10243. doi: 10.3390/ijerph181910243 (PMC8508568; doi:10.3390/ijerph181910243)
Supplement: Supplementary file 1 [file ijerph-18-10243-s001.zip › ijerph-1345270 Supplement S1_TDF_IJEPHR.pdf]

Supplement 1: The Theoretical Domains Framework integrated into the COM-B model (adapted from Cane et al 2012, pg. 15) [1]

**COM-B component**		**TDF domain**
Capability	Psychological	Knowledge
		Cognitive and interpersonal skills
		Memory, attention and decision processes
		Behavioural regulation
	Physical	Physical skills
Opportunity	Social	Social influences
	Physical	Environmental context and resources
Motivation	Reflective	Social/professional role and identity
		Beliefs about capabilities
		Optimism
		Intentions
		Goals
		Beliefs about consequences
	Automatic	Reinforcement
		Emotion

COM-B; capability, opportunity, motivation-behaviour, TDF; theoretical domains framework

1. Cane J, O'Connor D, Michie S. Validation of the theoretical domains framework for use in behaviour change and implementation research. Implement Sci. 2012;7.
