# Supplementary material for: Weight Loss Barriers and Dietary Quality of Intermittent and Continuous Dieters in Women with a History of Gestational Diabetes
Source: Int J Environ Res Public Health. 2021 Sep 29;18(19):10243. doi: 10.3390/ijerph181910243 (PMC8508568; doi:10.3390/ijerph181910243)
Supplement: Supplementary file 1 [file ijerph-18-10243-s001.zip › ijerph-1345270 Supplement S2_participant comments_IJEPHR.pdf]

Supplement 2: Participant comments on barriers to weight loss at baseline visit commencing 12-month RCT, linked to the TDF and COM-B model[1].

"Working in hospitality odd hours affects diet"
TDF Domains: Beliefs about capabilities; Environmental context and resources
COM-B components: Motivation – reflective; Opportunity - physical

"Lack of sleep"
TDF Domain: Memory, attention and decision processes
COM-B component: Capability - psychological

"I find it hard to allocate time and be organized"
TDF Domain: Skills
COM-B component: Capability - physical

"Studies"
TDF Domain: Beliefs about capabilities
COM-B component: Motivation - reflective

"Fussy eaters & busy life on weekends - not at home to eat"
TDF Domains: Social influences; Beliefs about capabilities
COM-B components: Opportunity – social; Motivation - reflective

"Arthritis in knees - can't walk far"
TDF Domain: Environmental context and resources
COM-B component: Opportunity - physical

"Physical restriction caused by increasing osteoarthritis in feet"
TDF Domain: Environmental context and resources
COM-B component: Opportunity – physical

“I find it hard to make time for exercise around work, kids and husband work shifts”

TDF Domains: Beliefs about capabilities; Environmental context and resources

COM-B components: Motivation - reflective; Opportunity - physical

“Need to be more organised with our daily meals at home”

TDF Domain: Skills

COM-B component: Capability - physical

“Motivation and tiredness”

TDF Domains: Emotion; Memory, attention and decision processes

COM-B Components: Motivation – automatic; Capability - psychological

“Time and energy”

TDF Domain: Beliefs about capabilities

COM-B component: Motivation - reflective

“Busy + erratic work schedule”

TDF Domains: Beliefs about capabilities; Environmental context and resources

COM-B components: Motivation - reflective; Opportunity - physical

“Work colleagues who love snacking at work”

TDF Domain: Social Influences

COM-B component: Opportunity - social

“M*aybe --> major depressive disorder / generalised anxiety disorder”*

TDF Domains: Emotion; Environmental context and resources

COM-B components: Motivation – automatic; Opportunity - physical

“Being around people who eat non diet food e.g. morning tea and meetings at work”

TDF Domain: Social influences

COM-B component: Opportunity – social

“Preparing kids snacks and meals & food as a reward in the evening”

TDF Domains: Social influences; Reinforcement

COM-B components: Opportunity – social; Motivation - automatic

“Needing to prepare separate food for me and the family”

TDF Domain: Social influences

COM-B component: Opportunity - social

“I'd like to lose weight (so not overweight and healthy) but it is hard to motivate myself when tired and a lot to do. Plus, when I try and eat more healthy food, I seem to put weight on.”

TDF Domains: Beliefs about capabilities; Memory, attention and decision processes

COM-B components: Motivation - reflective; Capability - psychological

“Hypothyroid (medicated)”

TDF Domain: Environmental context and resources

COM-B component: Opportunity - physical

“Shiftwork”

TDF Domain: Environmental context and resources

COM-B component: Opportunity - physical

“Time to exercise / go to the gym”

TDF Domain: Beliefs about capabilities

COM-B component: Motivation - reflective

“Preparing meals for kids at hoe and a 6yo fussy eater sometimes makes it hard”

TDF Domain: Social influences

COM-B component: Opportunity - social

“Immediate family + friends bringing foods into my home - Have often felt unsupported or sabotaged by them”

TDF Domain: Social Influences

COM-B component: Opportunity - social

“Motivation when not seeing results”

TDF Domain: Emotion

COM-B component: Motivation - automatic

“I have 2 sons with sensor processing problems which make meal time difficult and they tend to like unhealthy foods more”

TDF Domains: Environmental context and resources; Social influences

COM-B component: Opportunity – physical; Opportunity - social

“Consistency + time management biggest issues. Partner = shift work which is hard for scheduling gym. Finding consistent time to prep meals in advance”

TDF Domains: Beliefs about capabilities; Environmental context and resources

COM-B component: Motivation - reflective; Opportunity - physical

“Busy - lack of proper kitchen- no oven. Lack of bench space”

TDF Domain: Environmental context and resources

COM-B component: Opportunity - physical

“Suffering from depression / anxiety”

TDF Domain: Emotion

COM-B component: Motivation - automatic

[1] COM-B; Capability, Opportunity, Motivation-Behavior, TDF: Theoretical Domains Framework
